# Near-Infrared Spectroscopy Regional Oxygen Saturation Based Cerebrovascular Reactivity Assessments in Chronic Traumatic Neural Injury versus in Health: A Prospective Cohort Study

**DOI:** 10.3390/bioengineering11040310

**Published:** 2024-03-26

**Authors:** Alwyn Gomez, Izabella Marquez, Logan Froese, Tobias Bergmann, Amanjyot Singh Sainbhi, Nuray Vakitbilir, Abrar Islam, Kevin Y. Stein, Younis Ibrahim, Frederick A. Zeiler

**Affiliations:** 1 Section of Neurosurgery, Department of Surgery, Rady Faculty of Health Sciences, University of Manitoba, Winnipeg, MB R3E 0W2, Canada; 2 Department of Human Anatomy and Cell Science, Rady Faculty of Health Sciences, University of Manitoba, Winnipeg, MB R3E 0W2, Canada; 3 Department of Biosystems Engineering, Price Faculty of Engineering, University of Manitoba, Winnipeg, MB R3T 5V6, Canada; 4 Department of Biomedical Engineering, Price Faculty of Engineering, University of Manitoba, Winnipeg, MB R3T 5V6, Canada; 5 Undergraduate Medicine, Rady Faculty of Health Sciences, University of Manitoba, Winnipeg, MB R3E 0W2, Canada; 6 Centre on Aging, Fort Garry Campus, University of Manitoba, Winnipeg, MB R3T 2N2, Canada; 7 Division of Anaesthesia, Department of Medicine, Addenbrooke’s Hospital, University of Cambridge, Cambridge CB2 0QQ, UK; 8 Department of Clinical Neurosciences, Karolinksa Institutet, 171 77 Stockholm, Sweden; 9 Pan Am Clinic Foundation, Winnipeg, MB R3M 3E4, Canada

**Keywords:** traumatic brain injury, cerebrovascular reactivity, regional cerebral oxygen saturation, near-infrared spectroscopy, multimodal monitoring

## Abstract

Near-infrared spectroscopy (NIRS) regional cerebral oxygen saturation (rSO_2_)-based cerebrovascular reactivity (CVR) monitoring has enabled entirely non-invasive, continuous monitoring during both acute and long-term phases of care. To date, long-term post-injury CVR has not been properly characterized after acute traumatic neural injury, also known as traumatic brain injury (TBI). This study aims to compare CVR in those recovering from moderate-to-severe TBI with a healthy control group. A total of 101 heathy subjects were recruited for this study, along with 29 TBI patients. In the healthy cohort, the arterial blood pressure variant of the cerebral oxygen index (COx_a) was not statistically different between males and females or in the dominant and non-dominant hemispheres. In the TBI cohort, COx_a was not statistically different between the first and last available follow-up or by the side of cranial surgery. Surprisingly, CVR, as measured by COx_a, was statistically better in those recovering from TBI than those in the healthy cohort. In this prospective cohort study, CVR, as measured by NIRS-based methods, was found to be more active in those recovering from TBI than in the healthy cohort. This study may indicate that in individuals that survive TBI, CVR may be enhanced as a neuroprotective measure.

## 1. Introduction

The management of moderate-to-severe acute biomechanical neural injury, also known as traumatic brain injury (TBI), centers around the minimization of ongoing damage through the acute phase, known as secondary injury [1,2]. A key contributor to this secondary injury is the disruption of the brain’s ability to regulate cerebral blood flow (CBF) through microvascular vasomotion in response to fluctuations in arterial blood pressure (ABP) or cerebral perfusion pressure (CPP) [3,4]. This disruption in what is known as cerebrovascular reactivity (CVR) can result in periods of relative hypoperfusion or hyperperfusion, contributing to ongoing cerebral injury [5].

Significant advancements have been made in the role of CVR monitoring in the acute phase following moderate-to-severe TBI [6,7]. This includes associations with outcomes as well as work investigating CVR-guided management [8,9,10]. This progress has been largely driven by the development of continuous indices of CVR that allow for real-time monitoring of CVR at the bedside. The most prominent of these indices is the pressure reactivity index (PRx), which evaluates the correlation of intracranial pressure (ICP) as a surrogate for cerebral blood volume (CBV), with ABP as a surrogate for driving pressure [6,7]. Since the index is generated through a continuously updating Pearson correlation between ICP and ABP, values range from −1 to +1, with lower values indicating active CVR and higher values indicating a more vasopassive state [7].

PRx is ideally suited for monitoring CVR in critically ill TBI patients where invasive ICP monitoring is already indicated. However, its reliance on this invasive surrogate of CBV restricts its utilization in the chronic recovery phase or in healthy subjects. Fortunately, entirely non-invasive means of measuring CVR continuously have been developed [11,12]. The ABP variant of the cerebral oxygen index (COx_a) leverages near-infrared spectroscopy (NIRS) regional cerebral oxygen saturation (rSO_2_) as a surrogate for CBV and ABP as a surrogate for driving pressure. Like PRx, it utilizes a continuously updating Pearson correlation between rSO_2_ and ABP. As such, it also has values that range from −1 to +1, with higher values indicating a more vasopassive state [13]. Through novel techniques that leverage commercially available non-invasive continuous ABP finger cuff technology in conjunction with clinically utilized non-invasive rSO_2_ monitoring systems, COx_a can be measured entirely non-invasively [11,12]. This means that by combining these technologies, COx_a is not subject to the same limitations of PRx and can be utilized to evaluate CVR in populations that would otherwise be precluded from such studies. Recent work has also found COx_a to have similar statistical properties and a linear relationship to PRx [14,15]. Additionally, as rSO_2_ is a regional surrogate for CBV, COx_a has the potential to have improved spatial resolution compared to PRx, which is a global measure of CVR [16].

However, one existing knowledge gap surrounding this non-invasive NIRS-based CVR assessment method is its behavior in survivors long-term post-injury, and how this compares to a healthy control cohort. This prospectively conducted cohort study aims to examine CVR in a healthy group of volunteers by leveraging the non-invasive nature of COx_a. The role of age, biological sex, and hemispheric dominance on CVR are examined. Secondly, in a cohort of surviving patients recovering from moderate-to-severe TBI, CVR measured by COx_a is examined for trends through the recovery phase and compared to baseline values established through the healthy volunteer cohort.

## 2. Materials and Methods

### 2.1. Study Population and Data Collection

This prospective study consisted of two distinct cohorts. The first group consisted of adult (18 years or older) volunteers with no known neurological or cardiovascular conditions. These participants underwent physiological data recording during a single 30-min session in the resting awake state without any provocative testing. Data were collected between June 2020 and May 2023. Additionally, demographic information, such as age, biological sex, and handedness, were collected.

The second group consisted of adult patients that sustained moderate-to-severe TBI, based on an admission Glasgow Coma Scale (GCS) of 12 or less, for which they were admitted to the intensive care unit (ICU) at the Health Sciences Centre, Winnipeg, Manitoba, Canada between February 2020 and December 2022. Surviving patients underwent 30-min physiological data recording during standard outpatient follow-up at 3-, 6-, and 12-months post-injury. Data were collected between June 2020 and June 2023. Additionally, baseline demographic data and injury data were also collected [11].

**Figure 1:**
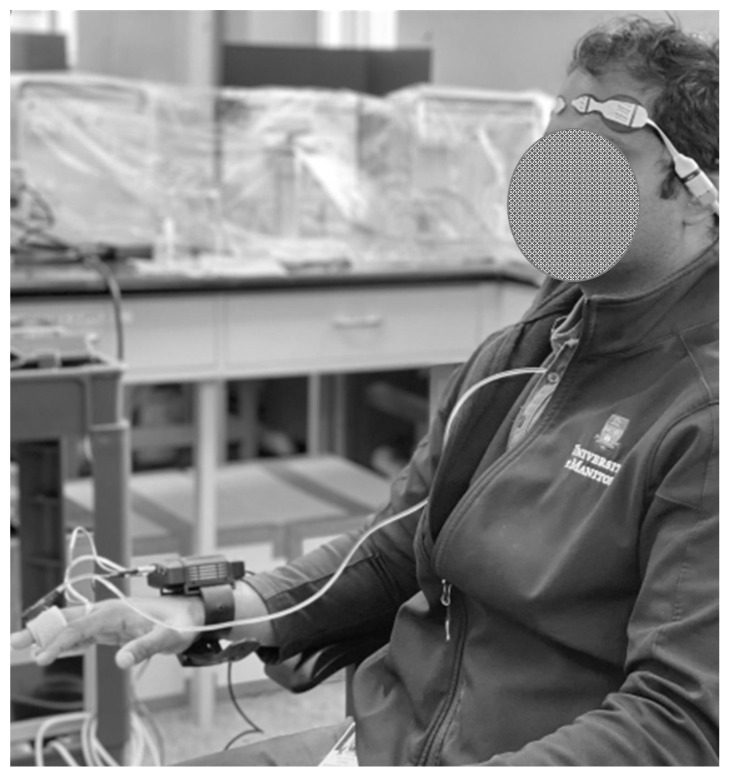
A demonstration of the setup of an entirely non-invasive collection of the arterial blood pressure (ABP)-based cerebral oxygen index (COx_a). Bilateral frontal lobe regional cerebral oxygen saturation (rSO_2_) was collected with sensor pads placed on the forehead (Covidien INVOS 7100, Covidien-Medtronic, Minneapolis, MN, USA) while arterial blood pressure was measured non-invasively thought a finger cuff (Finapres Nova, Palatijn, Enschede, The Netherlands).

Physiological data collection was identical between groups. ABP was collected continuously and non-invasively through a Finapres Nova finger cuff (Palatijn, Enschede, The Netherlands). Right and left frontal rSO_2_ was also collected continuously with the Covidien INVOS 7100 (Covidien-Medtronic, Minneapolis, MN, USA). The INVOS device emits wavelengths of light at 730 and 810 nm with two light detectors spaced 3 and 4 cm away from the emitter [17]. This arrangement allows the detector at 3 cm to provide an estimate of light absorbed by superficial tissues, while the detector at 4 cm provides an estimate of light absorption at a minimum tissue depth of approximately 1.3 cm [18]. The setup can be seen in Figure 1. ABP signals were digitized via an analog/digital converter (DT9803/DT9804/DT9826; Data Translation, Marlborough, MA, USA) with 100 Hz sampling while rSO_2_ was collected via digital data transfer at 1 Hz through serial output on the device. ICM+ software (Version 8.5, Cambridge Enterprise Ltd., Cambridge, UK) was utilized to record and time-lock physiological data streams. Artifacts were cleared manually utilizing ICM+ software by a trained research assistant. These artifacts were primarily due to loss of signal from NIRS sensor displacement or from disrupted ABP collection indicated by loss of ABP waveform.

### 2.2. Ethical Consideration

Full approval was received by the University of Manitoba Biomedical Research Ethics Board (B2020:019and B2018:103) and the Health Science Centre Research Impact Committee. For participants in both cohorts, full informed consent was obtained prior to study participation. Participants were able to withdraw from the study at any time.

### 2.3. Data Processing

Utilizing ICM+ software (Version 8.5), ABP and rSO_2_ signals were processed utilizing a 10-s non-overlapping moving average filter to remove high frequency signals not associated with vasomotion [19,20]. Next, COx_a was derived using a standard procedure of calculating a Pearson correlation between ABP and rSO_2_ over a 300-s window and updating the window every minute to obtain a minute-by-minute COx_a for both the right and left frontal channels [13]. This is a standard procedure for the derivation of continuous indices of CVR and is done to limit the influence of very low fluctuations in ABP and CBV that are unrelated to cerebrovascular vasomotion [6,19,20]. Minute-by-minute physiological data streams were then exported for each recording as comma separated value (.csv) files for further analysis.

### 2.4. Statistical Analysis

Utilizing R statistical software (Version 4.3.0, R Foundation for Statistical Computing, Vienna, Austria) and the *tidyverse* package, mean values for both right and left COx_a were determined for each recording. Within the healthy volunteer group, non-parametric statistical tests (Mann–Whitney U test and Wilcoxon Signed-Rank test, as appropriate) were conducted, along with standard linear regression to examine the role of hemispheric dominance, biologic sex, and age in CVR. For the chronic TBI cohort, non-parametric statistical tests were conducted to compare the first and last available COx_a measurements. Additionally, the side of cranial surgery, if performed in the acute phase, was utilized as a surrogate for the side of worse injury. As such, COx_a on the side of cranial surgery was compared to the non-surgical side for first and last available measurements. Similarly, COx_a values in the chronic TBI group were tested for statistical difference (Mann–Whitney U test) with the healthy volunteer group. For all testing, alpha was set to 0.05 and not corrected for multiple comparisons due to the exploratory nature of the analysis.

## 3. Results

### 3.1. Subject Demographics

**Figure 2:**
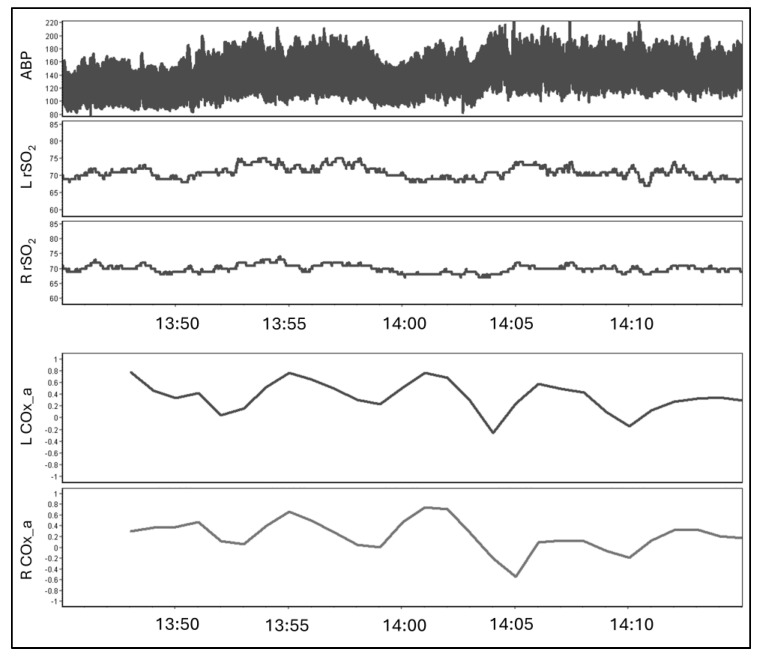
A 30-min sample of arterial blood pressure (ABP) sampled at 100 Hz and left and right frontal regional cerebral oxygen saturation (L rSO_2_ and R rSO_2_) sampled at 1 Hz. The bottom two series show the left and right ABP-derived cerebral oxygen index (L COx_a and R COx_a) updated on a minute-by-minute basis.

In total, 101 healthy volunteers were recruited with one 30-min recording for each subject. There were 29 chronic TBI patients for which at least one follow-up recording was available at 3-, 6-, or 12-months and a total of 65 30-min recordings were performed during follow-up. A simple processed minute-by-minute data recording can be seen in Figure 2. The full demographic data for the healthy volunteers and chronic TBI patients can be found in Table 1 and Table 2, respectively. Notably, the cohort of healthy volunteers was statistically younger than the cohort of chronic TBI patients (26 (IQR: 22 to 31) vs. 40 (IQR: 25 to 50), *p* < 0.001 Mann–Whitney U test).

### 3.2. Healthy Volunteers

**Figure 3:**
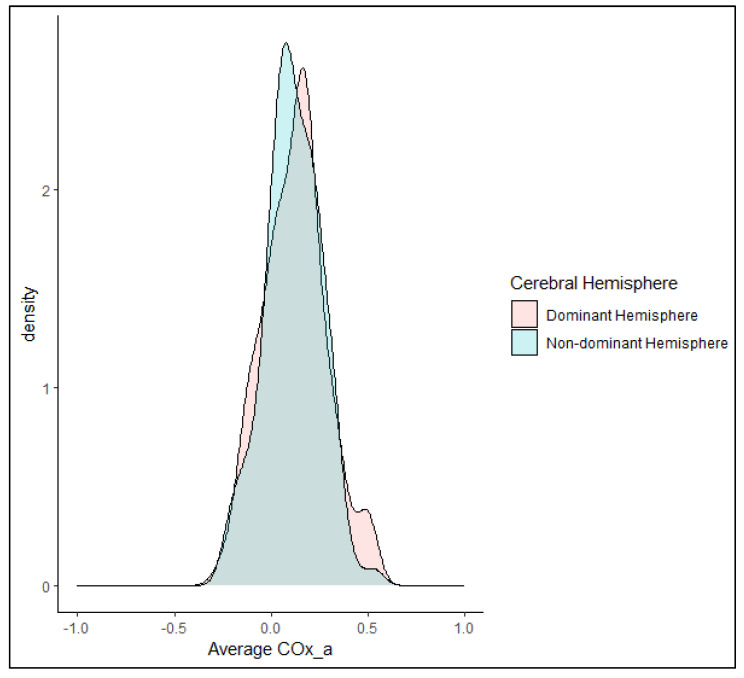
A density plot of the distribution of average arterial blood pressure (ABP)-derived cerebral oxygen index (COx_a) by cerebral hemisphere in the healthy volunteer cohort. Note the significant degree of overlap between groups, consistent with the lack of statistically significant differences between COx_a in dominant (rose) and non-dominant (blue) hemispheres.

Given that COx_a is a theoretical measure of regional CVR, an exploration of hemispheric difference was first examined. COx_a values in the dominant hemisphere were compared to that of the non-dominant hemisphere in each subject utilizing a paired sample Wilcoxon test. Handedness was used as a surrogate for hemispheric dominance for language. In the healthy cohort, nine subjects reported left handedness (right hemispheric dominance) and 92 subjects reported right handedness (left hemispheric dominance). No statistically significant difference in COx_a values was found between the dominant and non-dominant hemispheres (0.14 (IQR: 0.02 to 0.21) vs. 0.11 (IQR: 0.03 to 0.21), respectively, *p* = 0.500 Wilcoxon Signed-Rank test). Figure 3 shows the distribution of average COx_a values by cerebral hemisphere for the group of healthy volunteers. Given this result, left and right COx_a values were averaged for each subject for the remaining analyses.

**Figure 4:**
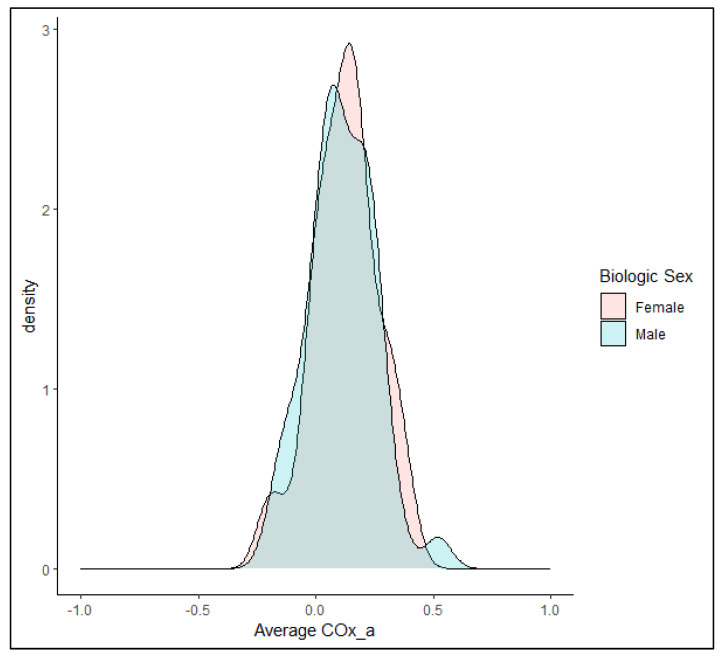
A density plot of the distribution of average arterial blood pressure (ABP)-derived cerebral oxygen index (COx_a) by biologic sex in the healthy volunteer cohort. Note the significant degree of overlap between groups, consistent with the lack of statistically significant differences between COx_a in females (rose) and males (blue).

Similarly, there was no statistically significant difference between CVR in males and females as measured by COx_a (0.10 (IQR: 0.03 to 0.20) vs. 0.12 (IQR: 0.04 to 0.20), respectively, *p* = 0.564 Mann–Whitney U test). Figure 4 shows the distribution of averaged COx_a values by biologic sex for the group of healthy volunteers. Additionally, age was not a significant univariate linear regressor for COx_a values in the cohort.

### 3.3. Chronic Traumatic Brain Injury Patients

**Figure 5:**
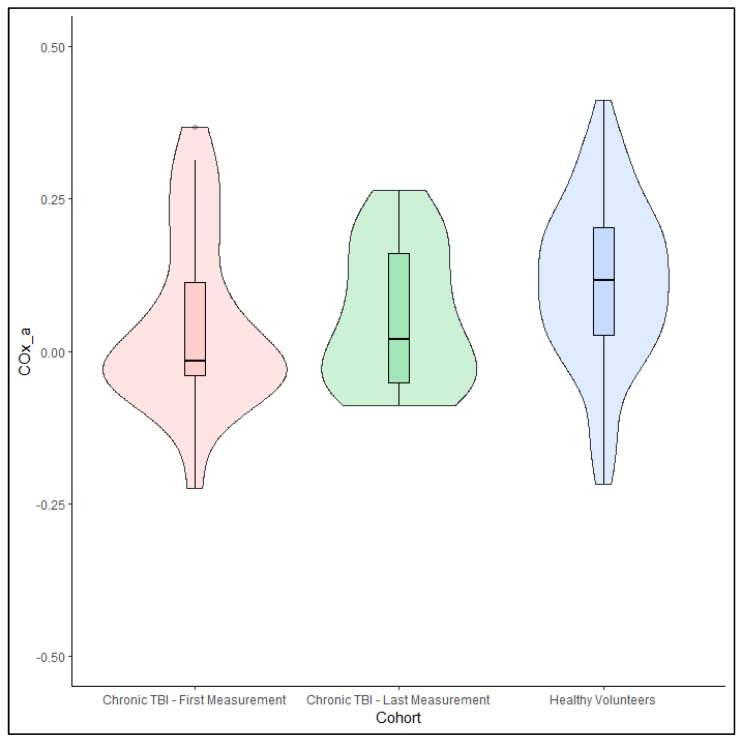
A violin plot of the distribution of average arterial blood pressure (ABP)-derived cerebral oxygen index (COx_a) by cohort. Note the significantly higher values of the healthy volunteer group (blue) compared to the first available (rose) or last available (green) measurement in the chronic traumatic brain injury (TBI) cohort; however, a high degree of overlap is still present.

Age was not found to be a significant univariate linear regressor for COx_a in either the first or last available follow-up in the chronic TBI cohort. Interestingly, COx_a was statistically lower in both the first available (−0.02 (IQR: −0.04 to 0.11), *p* = 0.005 Mann–Whitney U test) and the last available (0.02 (IQR: −0.05 to 0.16), *p* = 0.021 Mann–Whitney U test) follow up measurement in the chronic TBI group compared to the cohort of healthy volunteers (0.12 (IQR: 0.03 to 0.20)). While there was no statistical difference between COx_a at the first available measurement and the last available measurement, there was a trend for COx_a to be higher during the last available follow up (−0.02 (IQR: −0.04 to 0.11) vs. 0.02 (IQR: −0.05 to 0.16), respectively, *p* = 0.537 Wilcoxon Signed-Rank test). Figure 5 shows the distribution of COx_a values for these groups in a violin plot. In those that underwent cranial surgery, COx_a was not found to be significantly different between the side of surgery and the non-surgical side at either the first available measurement (−0.01 (IQR: −0.08 to 0.03) vs. 0.06 (IQR: −0.06 to 0.16), *p* = 0.497, Wilcoxon Signed-Rank test) or the last available measurement (0.05 (IQR: 0.00 to 0.16) vs. 0.03 (IQR: −0.06 to 0.16), *p* = 0.376, Wilcoxon Signed-Rank test).

## 4. Discussion

In this prospective cohort study, the non-invasive nature of COx_a was leveraged for the first time to examine the CVR in both a healthy group of volunteers as well as in a group of patients recovering from moderate-to-severe TBI. This was made possible by the novel, entirely non-invasive method of measuring COx_a. This study presents several interesting findings relating to both healthy subjects and chronic TBI patients.

First, by leveraging the improved spatial resolution of NIRS, hemispheric differences in CVR were able to be explored in a healthy population. There were no statistically significant differences in COx_a between the dominant and non-dominant frontal lobes examined through the NIRS sensors. While prior studies leveraging various modalities have identified hemispheric differences in the pathologic state, there is little evidence for differences in the healthy state [21,22]. It should be noted that no provocative testing was performed to augment potential cerebral activity in the presumed dominant hemisphere for speech. In a healthy resting state, hemispheric differences in CVR are likely minimal.

When assessing differences in CVR based on the biologic sex of the healthy volunteers, no significant difference was found. While the TBI cohort was not perfectly balanced between male and female participants in terms of numbers, both groups were well represented in this cohort. This is a departure from what has been observed in early studies examining sex differences in CVR in the acute TBI setting, where females were found to have worse CVR [23,24,25]. More recent studies in the acute TBI population, like this one, have failed to identify any difference in CVR between males and females [26,27]. It should be noted that those prior studies were limited by cohorts that were predominantly male. This was similar to the currently presented chronic TBI cohort, where the vast majority of patients were male, limiting the value of examining sex differences in this cohort. In a healthy state, CVR does not significantly differ between males and females.

Age was not found to be a significant regressor for COx_a in the healthy volunteers; however, the age distribution of this group was quite narrow with a distribution centered around a young age. Similarly, age was not a significant regressor for COx_a at either the first or last available visit of the chronic TBI cohort. It should be noted that, in both cohorts, the age distribution was very narrow and centered around a younger age. This may explain, in part, why these findings are in contrast to prior studies examining the effect of age on CVR in acute TBI, where CVR was found to be worse with age [8,23,28,29,30,31,32]. In the literature on healthy subjects, a recent study showed no age-related difference when CVR was evaluated with transcranial Doppler (TCD) [33]. Thus, it remains unclear if differences in CVR with age is a phenomenon isolated to the acute injury phase or if the age distribution of the present cohorts have limited detection of this effect over a wide age range.

Utilizing the side of cranial surgery as a surrogate for the side of worse cerebral injury, there was no statistical difference between COx_a on the surgical side versus the non-surgical side at either the first or last available measurement. The side of surgical intervention was felt to be the most objective indicator of the side of worse injury, however, given that less than half of the cohort underwent cranial surgery, these findings may be the results of an underpowered subgroup analysis.

Perhaps the most interesting finding of this study is that CVR, as measured by COx_a, was found to be more active at both the first and last available follow-ups in the chronic TBI cohort than in the healthy volunteer cohort. Additionally, CVR appeared to trend towards normalization between the first and last follow-up of the chronic TBI patients. This could indicate that in those that survive moderate-to-severe TBI, CVR is hyperactive in the recovery phase. This could be due to either a survival bias, in which those that survive this type of injury have more active CVR at baseline, or it could be a true adaptive mechanism of neuronal protection in the recovery phase of injury. The trend towards normalization seen in this cohort could also indicate that this is a mechanism that dissipates with time. In a recent study examining CVR leveraging TCD and provocative testing (sit-stand maneuvers), differences between a healthy group and a chronic TBI group were minimal. However, time since injury in that study was much longer, with a median time of 18 months since injury, which may support the notion of normalization over time [34].

An alternative hypothesis is that a reduced COx_a in the chronic TBI cohort may represent an inability of NIRS-based rSO_2_ to act as a surrogate for CBV. This might be due to artifacts from the prior craniotomy, cranioplasty, or from cerebral atrophy and gliosis following injury. In both instances, the rSO_2_ signal might not truly be that of viable cerebral tissue. This could result in an rSO_2_ signal with little variability that is completely decoupled from ABP changes and would manifest as a COx_a value close to zero. As the COx_a values in the healthy volunteers are above zero, this artifactual decoupling of rSO_2_ signals would give the appearance of a more active CVR state. It is also worth noting that while statistically significant, the difference between the two cohorts is not very large and the physiological relevance of this difference is unclear.

### 4.1. Study Limitations

There are a number of limitations that must be considered when interpreting the findings of this study. First is that in both groups the age distribution was somewhat limited, with few subjects being included with advanced age. This limits the ability of these cohorts to identify age-related variation in CVR. Similarly, the small number of female subjects in the chronic TBI cohort, while consistent with the epidemiology of the condition, limits evaluation of biologic sex differences in this pathologic state. Subject age was also significantly different between the chronic TBI cohort and the healthy volunteer cohort which may have impacted CVR in an unpredictable way.

While 29 subjects were included in the chronic TBI cohort, there were a significant number of missed follow-up sessions, with only 65 recordings available out of a potential 87. This limited the ability to fully examine the trajectory of COx_a over the course of recovery, with all three recordings available for less than half of the cohort. Additionally, those that did participate generally had quite good functional outcomes as measured by extended Glasgow outcome scale (GOSE). This means that the chronic TBI cohort was nearly fully recovered from their TBI. This type of sampling bias may have increased physiologic similarity between the healthy volunteer and chronic TBI cohorts. Further, the limited number of subjects in this study, while able to provide statistically significant findings, limits the ability to determine the clinical significance of such findings.

Additionally, the low temporal resolution of the NIRS device used in this study precluded the use of more sophisticated wavelet coherence techniques for evaluating CVR continuoulsy. These methods, however, are still in their early development and their advantages over time-domain–based indices of CVR are not fully understood [29,35]. Further, the limited evaluation of only the frontal lobes in the chronic TBI cohort would not fully evaluate CVR in the region of injury in those with cerebral insult in alternative locations. It should be noted, however, that popular contemporary measures of CVR, such as PRx, are also global measures of CVR with no better spatial resolution than COx_a [36].

Finally, rSO_2_ can be affected by a number of factors, beyond pressure-modulated arteriolar dilation and constriction, data for which were not collected during this study. This includes any factor that changes the regional concentrations of oxygenated hemoglobin (OxHgB) and total hemoglobin (HgB) values, such as systemic HgB levels and systemic oxygen saturation (SpO_2_). Metabolic factors, such as end-tidal carbon dioxide (ETCO_2_) and neuronal activity, and therefore the effects of neurovascular coupling, were not strictly controlled or measured. Additionally, as rSO_2_ is prone to interference, it is notable that no provocative testing, such as hyper- and hypoventilation, was performed to ensure that rSO_2_ signals were reflective of cerebral microvasculature, as assumed by COx_a.

### 4.2. Future Work

The findings of this study will need further validation and exploration. Future studies examining CVR utilizing COx_a would benefit from larger multicenter cohorts, with more age profiles and an even split in biological sex. This would better identify age and biological sex contributions to CVR in healthy volunteers and chronic TBI patients. The inclusion of provocative testing, such as cognitive testing and language testing may also elucidate hemispheric differences in CVR. Additionally, ETCO_2_ and SpO_2_ monitoring coupled with respiratory rate modulation would also help to ensure that rSO_2_ signals were reflective of cerebral microvasculature.

Additionally, utilizing NIRS devices with improved temporal resolution and more comprehensive cortical mapping, a fuller evaluation of CVR may be possible in both cohorts. This could be accomplished through leveraging wavelet coherence-based methodologies in combination with a complete spatial assessment of the brain [37].

Finally, while the evolution of COx_a was evaluated here in a chronic TBI population, it would be of interest to examine the outcome associations of COx_a in the chronic phase. Additionally, the relationship between CVR in the acute versus chronic phases should also be explored.

## 5. Conclusions

This prospective study leveraged novel techniques of non-invasively measuring CVR to evaluate various metrics in both healthy volunteers and chronic TBI patients. Within the cohort of healthy volunteers, CVR, as measured by COx_a, did not differ by biologic sex or the hemisphere of language dominance. There was no clear relationship between age and COx_a either; however, the demographics of the cohort were skewed to a younger population. Compared to the healthy volunteers, chronic TBI patients had more active CVR at follow-up with a trend towards normalization of CVR at last available follow-up, as determined by COx_a. Future studies will need to validate these findings in a larger, more distributed cohort.

## Figures and Tables

**Table 1:** Demographic Data of the Healthy Volunteers.

Demographic Parameter		Median or Number of Patients N = 101
**Age (IQR)**		26 (22 to 31)
**Biologic Sex**	***Male subjects (%)***	41 (40.6)
	***Female subjects (%)***	60 (59.4)
**Handedness**	***Right (%)***	92 (91.8)
	***Left (%)***	9 (8.9)
**Subject Average COx_a (IQR)**		0.12 (0.03 to 0.20)
**Subject Average ABP (IQR)**		101.5 (83.8 to 105.5) mmHg
**Subject Average rSO_2_ (IQR)**	***Right***	72.6 (66.2 to 79.1)%
	***Left***	73.7 (69.6 to 79.0)%

ABP = arterial blood pressure; COx_a = cerebral oxygen index based on arterial blood pressure; IQR = interquartile range; rSO_2_ = regional cerebral oxygen saturation.

**Table 2:** Demographic Data of the Chronic Traumatic Brain Injury Patients.

Demographic Parameter		Median or Number of Patients N = 29
**Age (IQR)**		40 (25 to 50)
**Biologic Sex**	***Male subjects (%)***	24 (82.8)
	***Female subjects (%)***	5 (17.2)
**Admission GCS (IQR)**		7 (4 to 8)
**Admission Pupils**	***Bilaterally reactive (%)***	22 (81.5)
	***Unilaterally reactive (%)***	5 (17.2)
	***Bilaterally unreactive (%)***	1 (3.4)
**Cranial Surgery Side**	***Right***	7
	***Left***	6
	***None***	16
**Marshall CT Classification**	***I (%)***	0 (0)
	***II (%)***	1 (3.4)
	***III (%)***	11 (37.9)
	***IV (%)***	6 (20.7)
	***V (%)***	11 (37.9)
	***VI (%)***	0 (0)
**Follow-up GOSE**	***3-Month***	6 (5 to6)
	***6-Month***	7 (5 to7)
	***12-Month***	1 (5)
**Recording Sessions**	***3-Month***	23
	***6-Month***	24
	***12-Month***	18
**Subject Average COx_a (IQR)**	***3-Month***	−0.02 (−0.04 to 0.06)
	***6-Month***	0.05 (−0.06 to 0.17)
	***12-Month***	0.02 (−0.04 to 0.11)
**Subject Average ABP (IQR)**		93.5 (83.8 to105.5) mmHg
**Subject Average rSO_2_ (IQR)**	***Right***	69.0 (64.6 to73.9)%
	***Left***	70.1 (65.5 to 73.7)%

ABP = arterial blood pressure; COx_a = cerebral oxygen index based on arterial blood pressure; CT = computed tomography; GCS = Glasgow coma scale; GOSE = extended Glasgow outcome scale; IQR = interquartile range; rSO_2_ = regional cerebral oxygen saturation.

## Data Availability

The raw data supporting the conclusions of this article will be made available by the authors, without undue reservation.
